# Biosensors and Diagnostics for Fungal Detection

**DOI:** 10.3390/jof6040349

**Published:** 2020-12-08

**Authors:** Khalil K. Hussain, Dhara Malavia, Elizabeth M. Johnson, Jennifer Littlechild, C. Peter Winlove, Frank Vollmer, Neil A. R. Gow

**Affiliations:** 1 Medical Research Council Centre for Medical Mycology, University of Exeter, Geoffrey Pope Building, Stocker Road, Exeter EX4 4QD, UK; d.malavia@exeter.ac.uk (D.M.); elizabeth.johnson@PHE.gov.uk (E.M.J.); 2 UK National Mycology Reference Laboratory (MRL), Public Health England South-West, Science Quarter Southmead Hospital, Southmead, Bristol BS10 5NB, UK; 3 Biocatalysis Centre, University of Exeter, The Henry Wellcome Building for Biocatalysis, Stocker Road, Exeter EX4 4QD, UK; J.A.Littlechild@exeter.ac.uk; 4 Department of Physics and Astronomy, College of Engineering, Mathematics and Physical Sciences, University of Exeter, Exeter EX4 4QD, UK; C.P.Winlove@exeter.ac.uk; 5 Living Systems Institute, University of Exeter, Stocker Road, Exeter EX4 4QD, UK; F.Vollmer@exeter.ac.uk

**Keywords:** biosensors, diagnostics, medical mycology, fungal biomarker, point-of-care devices, immobilization

## Abstract

Early detection is critical to the successful treatment of life-threatening infections caused by fungal pathogens, as late diagnosis of systemic infection almost always equates with a poor prognosis. The field of fungal diagnostics has some tests that are relatively simple, rapid to perform and are potentially suitable at the point of care. However, there are also more complex high-technology methodologies that offer new opportunities regarding the scale and precision of fungal diagnosis, but may be more limited in their portability and affordability. Future developments in this field are increasingly incorporating new technologies provided by the use of new format biosensors. This overview provides a critical review of current fungal diagnostics and the development of new biophysical technologies that are being applied for selective new sensitive fungal biosensors to augment traditional diagnostic methodologies.

## 1. Introduction

Fungal infections represent a very high burden to world health and necessitate a considerable resource requirement for medical intervention. Dermatophyte infections of the skin, hair and nails are among the most common ailments of humans, which can affect up to 20% of the world population at some time in their lives [1,2]. It has been estimated that more than one hundred million women suffer recurrent vaginal mucosal infections and well in excess of 3 million adults worldwide are sensitised to fungal allergens that cause considerable lung-associated pathology [3,4]. In addition to this high burden of superficial infection, allergy and associated morbidity, current estimates suggest that attributable mortality figures for invasive fungal infections, most commonly caused by *Candida* spp., *Cryptococcus* spp., *Pneumocystis jirovecii* and *Aspergillus* spp., result in between 1 and 1.5 million deaths per annum, which exceeds the death toll attributed to malaria, and is a similar number to deaths due to HIV and tuberculosis (1). Life-threatening fungal infections usually only occur in patients with profound immunosuppression or in those weakened or rendered more vulnerable to infection due to trauma, co-morbidity or necessary surgical or other medical treatments and interventions. Fungal pathology is complex and can result either from the ability of the fungus to cause tissue destruction or stimulate an uncontrolled inflammatory immune response (sepsis) that can contribute to morbidity and mortality. As medical interventions become more sophisticated, with more patients surviving previously fatal conditions due to trauma, disease or infection, the pool of potentially susceptible patients increases with a commensurate increase in the incidence of established and emerging fungal infection. In most cases, late diagnosis of systemic fungal disease is associated with a poor prognosis [5,6]. For this reason, the development of accurate, rapid, specific and sensitive diagnostic tests has become a medical imperative.

Despite the critical importance of making an early and accurate diagnosis, in the area of applied medical mycology research funding and investment in diagnostics has lagged well behind public and private investment in therapeutic interventions. In the UK, for example, as few as 1.9% to 2.8% of more than 6125 studies in infectious diseases, which were conducted between 1997 and 2020, was funding awarded to mycological research, and clinical research received only 12.7% of this allocation [7]. The fraction of this small percentage of funding that included research into diagnostics was not specifically considered in this published study, but it is likely that it would be less than a fifth of the 12.7%, suggesting that research in fungal diagnostics may receive less than 0.1% of government research funding in the UK. This seems an inappropriately meagre investment into what is clearly a critical medical need.

Although there have been several significant recent developments, many of the key diagnostic techniques used in front-line diagnosis of invasive fungal infection have not changed substantially in many years. A strong reliance is still made on microscopy and fungal culture in vitro, histopathology, radiographic and CT imaging, serology and antigen detection tests including the use of lateral flow devices [8]. Some of these diagnostic methods have the advantage that they can be developed into point-of-care tests that can be applied under circumstances where advanced bespoke mycological expertise, such as that available in national mycological reference centres, is not available. Increasingly, these foundation methodologies are being complemented by high-technology molecular-based alternative technologies ranging from the use of polymerase chain reaction (PCR) and DNA-sequencing-based approaches, to protein fingerprinting by matrix-assisted laser desorptionionization time of flight (MALDI-TOF) mass spectrometry [9]. The future of diagnostic technologies includes the development of multiplex diagnostics that do not require fungal culture and ideally could include the simultaneous analysis of other important parameters such as providing information about the drug resistance profile of the fungal pathogen. Recent experimental advances have been made in which ultrasensitive laser-based technologies can rapidly scan for specific biomarkers of fungal infection. This review surveys the range of options that are currently available and under development, making specific reference to new and evolving techniques that have the potential to create diagnostics that are highly sensitive and specific and can be undertaken rapidly without any requirement for isolation and culture of the unknown aetiological agent of infection.

## 2. Conventional Diagnostic Tools

Traditional laboratory-based diagnostic approaches in mycology include microscopy, histology, culture and serology [10]. Direct microscopy and culture from normally sterile or non-sterile sites on the body are routine and represent gold standard diagnostic tools in the detection of fungal infection. Various types of clinical samples, including saliva, urine, blood, cerebrospinal fluid (CSF), bronchoalveolar lavage fluid (BAL), sputum, other body fluids, swabs and tissue, can be used to detect fungal infection by direct microscopy and growth in fungal selective or indicator growth media such as Sabouraud agar, CHROMagar or blood agar [11,12,13,14]. Culture of fungi has its advantages since it may yield the infecting isolate for either phenotypic, proteomic or molecular identification and specific antifungal susceptibility tests [15]. Sub-culture onto CHROMagar™ *Candida*, which is a chromogenic substrate yielding different colony colours for some pathogenic yeast species, or its use as an initial isolation plate, can help to suggest the identification and more importantly can help to ensure that further tests are conducted on pure cultures, rather than mixed yeast species, a common reason for the failure of commercial identification methods. Recently, there has been a new development in this area with the introduction of CHROMagar™ *Candida* Plus, an agar which clearly differentiates the emerging pathogen *Candida auris*, a yeast responsible for multiple nosocomial outbreaks world-wide, from other pathogenic and commensal yeast species. In tests, this was able to differentiate four different clades of *C. auris* from 52 other pathogenic yeast species from 15 genera. It also has potential as a primary isolation medium to test patients for skin colonisation with this pathogen, a common precursor to blood stream infection, and thus facilitate the cohorting of colonised patients [16].

However, it is often not possible to obtain suitable clinical samples, and fungal growth frequently takes from 24–72 h and occasionally many weeks for adequate growth to occur. In particular, blood culture tests are much faster to reveal bacteraemia than candidaemia, which often takes 2–6 days, and fungaemia is often not present in mould infections with the notable exception of infections with *Fusarium, Scedosporium, Aspergillus terreus* and a few other rare mould infections that are spread by haematogenous dissemination. Therefore, faster biomarker and molecular detection methods have been developed that can be applied directly to serum or other clinical samples, often negating the need for attempted culture. Moreover, the sensitivity of the culture method can also depend on sample collection, transportation and preservation and can be confounded by contamination [14,17,18].

Direct microscopy has been an important diagnostic compliment to obtaining a fungal culture and can often be used to make a preliminary diagnosis within hours of taking a sample. It can be applied to many sample types including tissue biopsies, CSF, BAL, other body fluids, sputum and swabs. Microscopy, can reveal distinctive features of fungi morphology that can be used for provisional identification, thereby providing immediate results. Evidence of fungal cell structures visualised in tissue biopsy specimens by either direct microscopy, cytology or histopathology is considered proof of invasive infection [19]. For example, septate moulds such as *Aspergillus fumigatus* can be distinguished from the pauci-septate Mucorales. The identification of polymorphic species such as *Candida albicans* growing as yeast or hyphae and the encapsulated yeast *Cryptococcus* are also easily revealed. Additionally, staining solutions such as Calcoflour White, Blankophor, Gram staining, India ink and a library of antibody-based immunostains can aid in visualisation of morphological properties of fungi thus enabling accurate diagnosis [20,21,22,23,24]. Calcoflour White is commonly applied to tissue samples to detect fungal elements such as hyphae, pseudohyphae and yeast cells, whereas India ink and Gram staining is recommended for the specific detection of Cryptococcal infections because the dye particles are excluded by the gelatinous capsule creating a visible halo around yeast cells that are suspended in a dark colloid of dye [25,26,27]. Automated microscopic examinations have been devised that have recently been applied to faecal samples to detect fungi that may be associated with gut diseases, although, classically, faecal samples have not been considered as good samples for mycological diagnosis due to the frequent carriage of commensal yeast. A two-step system based on artificial neural networks (ANNs) processes, identifies and quantifies different types of fungal cells in microscopy images based on set parameters. While the system corrects for some common observation errors and significantly enhances throughput, it is limited to the parameters set by the operator and potentially can miss observations that experienced observers would identify [28].

Another development involves combining molecular diagnostics and microscopy such as in PNA-FISH (peptide nucleic acid fluorescent in-situ hybridisation) [29,30]. PNA-probes consist of a fluorescent molecule attached to oligonucleotide bases that bind to species-specific ribosomal RNA [31]. Positive cultures generate fluorescent cells that can be detected by microscopy. The FDA (Food and Drug Administration) has approved a Yeast Traffic Light PNA-FISH kit that accurately identifies *Candida* species in 96% of tested blood cultures [32,33]. Devices that deploy magnetic bead traps have been used to capture low numbers of microbial pathogens in blood samples [33] and may help to increase the sensitivity of direct microscopical techniques when the number of free circulating fungal cells is low.

Under the umbrella of microscopy-based diagnostics, histological examination is a broadly and widely used technique used to detect fungal cells in tissues, and the finding of fungal elements constitutes proven fungal infection [19,34]. Tissue histology can also provide indication of the host immune response to invading fungi by observation of infiltrating white cells. Routine staining techniques include hematoxylin and eosin (H&E), Grocott (methanmine) silver (GMS) stain, Fontana–Masson stain, Ziehl–Neelsen stain and Periodic acid-Schiff (PAS) [35,36,37]. For a detailed review on the application of histology in the diagnosis of fungal infections, see Guarner et al. (2011). Furthermore, guidelines on the detection of fungi in histological samples have also been published [38,39]. Histology provides relatively rapid diagnosis and is particularly useful where live cultures cannot be obtained. However, biopsy for histology from sterile body sites often involves highly invasive procedures and may not be possible in thrombocytopenic or otherwise severely ill patients. Furthermore, similarities in fungal morphology and the diversity of clinical manifestations of different fungi, may require supporting confirmation using more specific diagnostic tools. For example, *Aspergillus* species are often difficult to differentiate from other hyaline, septate causes of hyalohyphomycosis and even some dematiaceous fungi using H&E staining alone. Immunohistochemistry (IHC) can provide greater specificity as it utilises antibodies that bind to species-specific fungal antigens [40,41]. However, wide-spread application of IHC is hindered by limitation in the number of commercially available species-specific antibodies and a high degree of cross-reactivity of some antibodies between some species so they are not widely employed in the clinical setting [42]. More commonly, specific identification of fungal elements is undertaken by the PCR amplification of fungal DNA with *Aspergillus*-specific, *Candida*-specific, mucoraceous mould-specific or panfungal primers, which can be undertaken on formalin-fixed or fresh tissue but has greater sensitivity on the latter. In the case of panfungal primers, the resulting product can then be directly sequenced for specific identification as discussed later in the section on molecular detection methods.

Imaging/Radiology using X-rays, high-resolution computed tomography (HRCT) and magnetic resonance imaging (MRI) can provide essential diagnostic evidence suggestive of invasive fungal infection. Although radiology does not allow for accurate identification of the causative agent or even allow for definitive diagnosis of a fungal aetiology, it does provide indications of the type and extent of infection to inform appropriate lines of treatment and can be useful for guiding biopsy sampling. Moreover there are some features that can be more suggestive of a fungal aetiology, for example, the presence of large nodules (>1 cm) or perinodular halos in chest radiographs can be indicative of angioinvasive fungal infections, and a reverse halo accompanied by rapid tissue invasion or multiple (≥10) nodules and pleural effusion can be suggestive of mucoraceous mould infection [43,44,45]. Diagnosis of *Pneumocystis* pneumonia can also be suggested by specific “ground-glass densities” in a chest radiograph, where the lungs appear white with vascular markings [46,47]. MRI scanning is particularly helpful in detecting ring-enhancing lesions and other features associated with the diagnosis of fungal infections of the CNS, bulls-eye lesions in liver and spleen tissue and fungal balls in kidney tissue [48].

Serology is a long-established and widely used method of detection of fungal infections. Diagnosis is achieved by identification of proteins usually antibodies in blood or saliva [49,50] using assays such as immunodiffusion (ID), counter-immunoelectrophoresis (CIE), Enzyme-Linked ImmunoSorbent Assays (ELISA), complement fixation (CF), lateral flow assays, radio-immunosorbent assays (RIA) and agglutination assays [51,52]. These techniques allow for the detection of circulating antibodies or fungal antigens depending on the test design. It should be acknowledged that antibody testing will only be helpful in patients able to mount an antibody response such as those with chronic infections including aspergilloma and endocarditis and also those otherwise healthy individuals with acute or chronic infections due to endemic dimorphic pathogens such as coccidioidomycosis and histoplasmosis. Antigen testing will be more helpful in patients with neutropenia or other conditions affecting humoral or cellular immunity [53]. For example, testing for the fungal antigens galactomannan, mannan and β-D-glucan in serum samples and testing for galactomannan in BAL samples are among those routinely performed serological tests for the detection of infections due to *Aspergillus, Candida* and some other mould infections in immunocompromised patients [18,54,55,56,57]. Common diagnostic tests for antibody detection such as, CF and ID are useful markers of infection with histoplasmosis and other endemic dimorphic pathogens, chronic aspergillosis and candidosis. These tests can be qualitative or semi-quantitative in which a titre is determined, providing valuable information on fungal burden that can inform antifungal therapeutic strategies and act as prognostic indicators. Some serological tests offer high sensitivity but there may be caveats that need to be considered. Cross reactivity has also been reported that compromises test specificity [58]. Antibody responses to infection may take 4–8 weeks to become detectable in peripheral blood making early diagnosis difficult. Hence, appropriate titre cut-off values are important to avoid false negative results particularly in the context of early stage infection [59,60]. Regardless of the drawbacks, serological tests are minimally invasive, inexpensive and provide rapid results that usefully inform clinical decisions.

## 3. Galactomannan Detection

Galactomannan (GM) is one of the most common biomarkers for the detection of *Aspergillus* infection and was one of the first to be commercially developed. GM is a 20 kDa polysaccharide located in the outer cell wall layer of *Aspergillus*, *Penicillium* and certain other fungal species and is shed from fungal hyphae during growth. Platelia™ *Aspergillus* Enzyme Immunoassay (EIA) (Bio-Rad, Marnes-la-Coquette, France) is a commercially available kit based on a one-stage immunoenzymatic sandwich micro-plate assay that detects galactofuranosyl-containing molecules using a rat monoclonal antibody directed at *Aspergillus*, although there are cross-reactions with some other fungal groups including *Fusarium, Geotrichum, Histoplasma*, *Paecilomyces*, *Penicillium,* and *Rhodotorula.* Moreover, there may also be cross reactions with galactomannan resorbed from the intestinal tract in patients with severe mucocitis, and certain batches of antibiotics have been shown to contain galactomannan leading to serum positivity in the absence of aspergillosis. In invasive aspergillosis, the highest concentrations of GM are released in the terminal phases of invasive disease and certainly after angioinvasion had occurred; thus, the test may lack sensitivity for early diagnosis [61,62]. Serum GM can be useful in predicting the outcome and assessing the response to antifungal therapy, but providing a higher cut-off index value is used it has been demonstrated that the galactomannan test conducted on BAL fluid is more sensitive and specific as an early diagnostic test for pulmonary aspergillosis [63]. It is generally considered that the galactomannan test displays optimal sensitivity and specificity when combined with other tests.

## 4. Mannan

Mannan is a major component of the yeast cell wall, and tests developed specifically to detect *Candida* infection include commercially available tests to detect mannan or mannoproteins, such as the Platelia™ *Candida* Ag Plus EIA (Bio-Rad, Marnes-la-Coquette, Paris, France) and the CandTec latex agglutination test (Ramco Laboratories, Stafford, TX, USA). There are two confounding factors: one is that *Candida* spp. are common human commensals and heavy colonisation can cause positive results in serum, and the second is that *Candid*a mannan is rapidly cleared from the circulation, so frequent testing is important. Repeated high *Candida* mannan tests can help to confirm chronic candidosis, usually seen in immunocompromised patients with consistent radiology revealing bulls-eye lesions in the liver and/or spleen. The best results generally seem to be achieved with a combination of mannan antigen and antimannan antibody detection (Platelia™ *Candida* Ab Plus; Bio-Rad) [54,64].

## 5. β-(1,3)-D-Glucan

The main structural polysaccharide components of the cell wall of fungal pathogens are glucan, chitin, and mannan. Of these, β-(1,3)-D-glucan is the most important and abundant polysaccharide component of many cell walls and is a common component of the cell walls of most pathogenic fungi [65,66]. Fungitell^®^ is one of the commercially available assay kits capable of detecting and quantifying the presence of β-(1-3)-D-glucan in serum and cerebrospinal fluid using a colorimetric method. Although it is generally considered a sensitive, non-specific pan-fungal test there are certain fungal groups which produce less (1-3)-β-D-glucan such as *Cryptococcus* spp. *Blastomyces* spp. and the mucoraceous moulds so will not be detected [67]. Detection of high beta-glucan levels is often encountered during *Pneumocyctis jirovecii* infection thus lack of detection is helpful in excluding this diagnosis [68]. Further, the conformational behaviour of linear oligo-β-(1-3)-D-glucosides was studied using NMR experiments and molecular modelling [69]. This theoretical study revealed that conformational properties of disaccharide fragments depended on neither their position in the chain nor the length of the chain. Interestingly, monoclonal antibodies were developed specifically recognizing β-(1-3)-D-glucan using hybridoma technology antigen. The developed antibodies interacted with species from *Aspergillus*, *Candida*, *Penicillium* genera and *Saccharomyces cerevisiae*, but not bacteria [70].

## 6. *Cryptococcal* Capsular Polysaccharide

One of the earliest fungal biomarkers to be commercially developed was the latex agglutination kit (IMMY Immuno-Mycologics, Norman, OK, USA) for the detection of the cryptococcal capsular antigen glucuronoxylmannan. For several decades, this test has proved invaluable in the early diagnosis of cryptococcal meningitis or other systemic infection and can be conducted on serum or CSF with sensitivity and specificity > 90% often reported [71]. Tests have to be conducted on neat and 1:10 dilutions to allow for a prozone effect at high concentrations of the antigen. More recently, the same company has developed a lateral flow device (LFD) for the detection of the same capsular antigen and this has shown great promise as a point-of-care test with a reported sensitivity and specificity of 93% and 100%, respectively [72]. It is especially useful in less developed countries due to its low cost and long shelf-life even at ambient temperature and although it is usually conducted on serum samples, thus requiring centrifugation, or on CSF, recent studies have demonstrated its utility when used with direct finger-stick blood samples producing 100% concordance between whole blood, serum and plasma [73]. This is the first truly point-of-care test (POCT) for detecting a fungal infection.

## 7. Point-of-Care Tests (POCT) in Fungal Diagnosis

A point-of-care test (POCT) is defined by the College of American Pathologists as ‘testing that is performed near or at the site of a patient with the result leading to a possible change in the care of the patient.’ POCTs are usually performed with minimal equipment by individuals that have not had laboratory training such as physicians, nurses, nursing assistants and sometimes even the patients themselves; these currently include such tests as home pregnancy tests and blood glucose monitoring. POCT diagnostics have great potential for front line interventions in the treatment of disease and can be split into those that require little equipment and are simple to perform which makes them ideal for use in areas of the world lacking sophisticated laboratory equipment, and those that although they make use of sophisticated techniques are rapid to perform and can be miniaturised and run directly on untreated blood, urine, or other body fluids directly at the patients bed-side, these are often referred to as ‘lab on a chip tests’ (LOC) [64]. Although such devices would have high acquisition costs, they could also be utilised in resource-limited environments. The World Health Organization comments on the desirability for them to be affordable, sensitive, specific, robust and user friendly [74,75]; portability would also be an essential attribute.

Currently in the field of medical mycology it is the lateral flow devices with techniques based on immunochromatography that show the greatest promise and indeed on occasion are already employed as POCTs. As previously described, there is a commercial lateral flow device for the detection of cryptococcal capsular antigen (IMMY Immuno-Mycologics, Norman, OK, USA) which can be used on fingerstick bloods and has revolutionised the diagnosis of cryptococcal meningitis in less developed countries where cryptococcal disease is a major cause of mortality within the HIV-infected populations [73]. There has also been commercial development of a lateral flow device for the detection of *Aspergillus* antigen (OLM diagnostics, Newcastle-upon-tyne, UK, USA). This was initiated by Thornton and colleagues (2008), who developed a specific monoclonal antibody (mAb JF5) that targets an early germ tube specific glycoprotein (JF5) of *Aspergillus* species [76]. Detection of this antigen in human serum or BAL samples is indicative of active invasive infection and can be detected at an early stage of infection. An added advantage of this test is that it will not detect fungal spores, as it requires the cells to germinate and start to invade tissues before the target glycoprotein is synthesised; thus it is much less susceptible to false positive results than other *Aspergillus*-specific diagnostic tests due to the confounding presence of fungal spore contamination. The device can be used with serum but can also be applied to BAL samples for the diagnosis of pulmonary aspergillosis, which means it could be employed in bronchoscopy suites as near-patient testing. Studies comparing results with the lateral flow device, galactomannan and beta-glucan detection as well as *Aspergillus*-specific PCR tests conclude that it is suitable as a POCT and is a useful adjunct to other biomarker tests in the diagnosis of chronic pulmonary aspergillosis and invasive aspergillosis [77,78]. Galactomannan-like antigens were also targeted using IgM mAb476 to detect *Aspergillus* infection [79] (Table 1). Several companies have introduced β-(1,3)-D-glucan detection kits, including Fungitec G-Test MK (Seikagaku) and Fungitell (Associates of Cape Cod), the kinetic turbidimetric β-Glucan Test Wako (Wako Pure Chemical Industries) and the endopoint chromogenic B-G Star kit (Maruha). These differ somewhat in their dynamic range and positive cut offs [80]. As mentioned above, the CHROMagar™ *Candida* Plus agar could also be utilised as a near-patient surveillance test with patient skin swabs being sub-cultured directly onto the agar to detect colonization with the pathogenic yeast *Candida auris*, although this agar would then require incubation for 48 h before yielding the results.

## 8. Nucleic Acids Based Diagnostics

Various in-house polymerase chain reaction (PCR) tests have been developed and employed in fungal diagnostics in a wide range of settings. Molecular tests include conventional PCR, nested PCR, real-time PCR (RT-PCR), PCR based on ITS regions and rDNA, PCR-ELISA, multiplex PCR, and direct DNA sequencing. This range of methodologies brings obvious benefits in terms of the specificity of diagnosis, as primers can be designed to detect specific pathogens; however, there are challenges in terms of sensitivity and reproducibility, in particular in the generation of false negative results. Although conventional PCRs are rapid and can offer advantages in sensitivity, there are no standard FDA-approved protocols and hence results can be subject to lab-to-lab variability [8,81,82,83]. This reality is well recognised even in experienced molecular labs where PCR methodologies are used routinely and there have been efforts to standardise various aspects of testing with recommendations from the European *Aspergillus* PCR Initiative (EAPCRI)/ISHAM (International Society for Human and Animal Mycology) working group [84]. A recent publication by the American Thoracic Society recommended PCR to confirm *Aspergillus* infection in immunocompromised patients using blood or serum samples [85]. Modified “nested PCR” protocols have been established for improved specificity and sensitivity. This is achieved by subjecting samples to two consecutive PCR reactions using two sets of primers, thus allowing for the detection of fungal DNA as low as 1 fg with 100% specificity; however, this is dependent on sample type and concentration and is extremely vulnerable to contamination [86]. Endoscopic sinus surgery specimens were found to be particularly unsuitable for this technique perhaps due to contamination with high levels of environmental fungi in the nasal cavity [87].

One of the drawbacks of organism-targeted PCR is the requirement of a hypothesis to be made of the nature of the suspected aetiological agent. An alternative strategy is to undertake a preliminary diagnosis to confirm that the sample is positive in a pan-fungal DNA amplification which is then further tested using target-specific primers designed to confirm the presence of specific fungal organisms. A draw-back of this approach is that panfungal primers are by their nature designed to detect conserved sequences present throughout the kingdom and are thus less sensitive than specific PCR primers. Several commercial PCR tests exist for *Aspergillus* and *Candida* and there are also commercial tests for *Pneumocystis* and mucoraceous moulds. A recent evaluation found all the commercial *Aspergillus* PCR tests to have comparable sensitivity and specificity on serum samples but noted that sensitivity was significantly lower on serum than on respiratory samples. Two tests— the MycAssay *Aspergillus*^®^ (Myconostica, Cambridge, UK) and the AsperGenius^®^ (PathoNostics B.V., Maastricht, The Netherlands) assays—were recommended for routine PCR-detection of *Aspergillus* spp. DNA in respiratory samples [88]. Two of the kits, AsperGenius^®^ and MycoGenie^®^ (Ademtech, Pessac, France), have the useful additional attribute of detecting the resistance markers TR_34_/L98H and TR_46_/Y121F/T289A associated with the environmental azole resistance now being reported more widely in *A. fumigatus* isolates from patients [88,89]. All can be used directly on patient samples but could also be applied to panfungal products. Alternatively, the product of a pan-fungal PCR can be sequenced and matched to databases to reveal the identification with the proviso that care should be taken when interrogating public databases due to the presence of erroneous entries. Pan-fungal PCR amplifications have targeted ribosomal DNA for 18S, 5.8S and 28S ribosomal RNA subunit genes. These ribosomal genes exist in close proximity in the genome and are separated by internal transcribed regions (ITS1 and ITS2) which differ between fungal species and can be exploited to achieve an identification at the species level. These regions have therefore proved very fruitful in developing tests to identify fungi isolated from clinical samples [90]. These PCR tests are highly sensitive, so care must be taken when being used for direct diagnostic purposes to consider possible confounding positives originating from colonizing or contaminating organisms in patient samples [91]. Real-time PCR (RT-PCR) allows for the quantification of fungal burden, thereby helping to differentiate between the presence of actively growing fungi and contaminating fungal spores. Additionally, multiplex PCR has been developed, which allows for the rapid and simultaneous amplification of several targets by using multiple primer sets in the same sample [92]. In a recent study, a multiplex PCR assay showed 100% sensitivity and 94.1% specificity in the detection of *Candida* spp. in samples acquired from 58 patients with candidaemia [93]. PCR-ELISA is a less commonly employed molecular method, but it does have some unique qualities. These tests quantitatively detect digoxigenin-labelled PCR products that hybridize to target-specific probes immobilised on ELISA plates [94,95]. However, sensitive and quantitative, PCR-ELISA methods have been increasingly replaced by RT-PCR due to its inherent higher accuracy.

PCR-based diagnostics are also subject to some common limitations. The sensitivity of PCR is dependent on the type, complexity and processing of samples. For example, the sensitivity of PCR is significantly reduced in formalin-fixed paraffin-embedded samples as fixation damages nucleic acids, thus inhibiting the PCR reaction. However, the technique often does prove helpful on such samples, especially if fresh tissue samples are not available [96]. Secondly, the design and choice of PCR primers affects the outcome of a PCR reaction. Primers that have reduced target specificity may increase the likelihood of false-positive results. Alternately, if primers bind weakly to the target, this may lead to false-negative results. Furthermore, it is estimated that up to 20% of the unedited sequences in the GenBank have incorrect lineage designations which will affect identification of fungal species that depend on DNA sequencing of DNA and primer design and hence the efficiency of PCR amplification [97].

A new, FDA-approved, commercial platform, the T2 Magnetic Resonance (T2MR) (T2 Biosystems), is being evaluated in several centres for its utility in detecting the five most common pathogenic *Candida* species in blood samples, alongside a large array of other blood stream pathogens. This automated qualitative molecular diagnostics system utilizes T2 magnetic resonance (T2MR) in which particles coated with target-specific agents form clusters when they encounter the target pathogen in clinical samples. Complexes formed in this way alter the micro-environment of water molecules around the target which is then quantified in the presence of a magnetic field. Although acquisition costs are high and individual sample costs are significant, the system can detect low numbers of *Candida* cells within 5 h without prior sample purification steps [98].

A recent advance in fungal diagnostics is the application of MALDI-TOF MS (Matrix-assisted laser desorption ionization-time of flight mass spectrometry). This technique identifies species-specific fungal peptides instead of nucleic acids. Of the commercially available MALDI-TOF MS platforms, Bruker Biotyper (Germany) and Vitek MS (France) are approved by the FDA. Various studies have reported sensitivity of this diagnostic technique in identification of various fungal species including yeast and mould isolates [99,100,101]. In a recent survey, MALDI-TOF MS accurately identified 92.7% of tested fungal species [102]. The technique offers a rapid and accurate identification of closely related species and even uncommon pathogenic fungi such as *Paecilomyces* species [103,104]. However, while intact cells have been subjected to MALDI-TOF MS analysis, cell lysates are preferred and sample preparation can be particularly challenging for moulds and spores of fungi due to their robust cell walls. Additionally, the quality of reference database and analysis methods (e.g., spectrum scoring) can influence the efficiency of fungal identification. These databases and methods can vary between labs as well as between MALDI-TOF MS platforms. While reference databases are constantly updated and can provide up to 99% accuracy in the identification of fungal species, a number of these are still based on in-house libraries which are not always available publicly [105,106]. To date, in the field of medical mycology, this technique has been used predominantly as an identification tool for yeast species and in centres that have invested in the technology that has replaced phenotypic carbohydrate assimilation and other phenotypic tests, thus reducing the identification of common yeast isolates from 48 h to as little as 30 min [107]. Recent developments also suggest some utility in the detection of antifungal drug resistance [108]. Latterly, many mould species isolated from clinical specimens have also been added to databases, thus increasing the clinical utility of this approach [109]. However, there have also been direct applications to clinical samples, usually yeast detection in blood samples, in an attempt to reduce the time required for culture, thereby allowing for earlier diagnosis and identification of the infecting organism facilitating the prompt administration of appropriate antifungal therapy [110,111,112].

## 9. Biosensors for Fungal Detection

Fungal diagnostic research is likely to benefit significantly from current and future advances in biosensor technology that deploy a raft of methodologies that as yet have seldom been applied in the context of medical mycology. It is likely that biosensor technologies will play an increasingly important role in the diagnosis and monitoring of all infectious diseases and that they will prove to have significant utility in the early detection of fungal infection. Moreover, biosensors also offer opportunities for continuous monitoring of analytes that may help to assess response to treatment.

Sensors are analytical devices that are able to transform either chemical, physical or biological information into useful analytical signals. According to the International Union of Pure and Applied Chemistry (IUPAC), biosensors are defined as “integrated receptor-transducer devices, which are able to provide selective quantitative or semi-quantitative analytical information using a biological recognition element” [113]. Typically, sensors and biosensors consist of three component parts: (1) a recognition element (which distinguishes a particular analyte or a group of analytes), (2) a transducer (that produces an electrical signal), and (3) a signal processor. Biosensor technology is an active area of current research, often highly interdisciplinary in nature, involving biologists, analytical chemists, physicists and biophysicists.

In the next sections, we outline the general principles within which biosensors are designed and operate. We describe examples of how these methodologies are currently utilised, and what future opportunities exist for the design of next-generation fungal diagnostic strategies.

## 10. Working Principal of Biosensors

**Figure 1:**
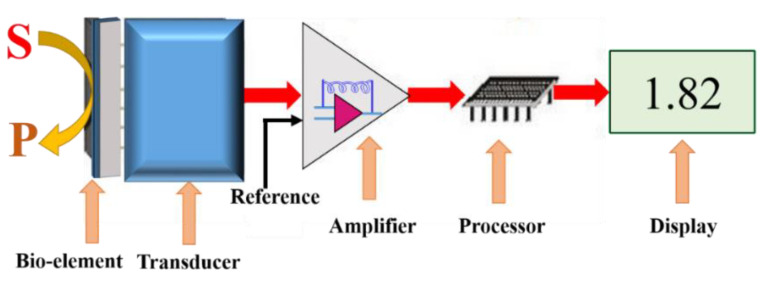
Schematic and working principle of a biosensor used to measure the conversion of a substrate (S) to a product (P) on a bio-element surface.

The recognition and transducer components of biosensors work together to produce a measurable signal. The role of a biological recognition system is to translate information from the biochemical domain, usually an analyte concentration, into a chemical or physical output signal with a defined sensitivity and ultra-high degree of selectivity. The transducer part of the sensor serves to transfer the signal from the output domain of the recognition system to the electrical output domain. High affinity binding transducers such as antibodies may give good sensitivity but binding may be irreversible and generate a “one shot” detector. In contrast, lower affinity may allow continuous monitoring of changing levels of analyte. Therefore, transducers convert defined chemical concentrations and binding events into electrical signals. The working principle of the biosensor is illustrated in the schematic below (Figure 1). Depending on the type of the signal generation system or transducer, biosensors are classified as: (i) electrochemical, (ii) optical, (iii) piezoelectric, or (iv) thermometric.

### 10.1. Electrochemical Biosensors

Electrochemical biosensors are integrated receptor-transducer devices that are capable of providing selective quantitative or semi-quantitative analytical information utilizing the properties of a biological recognition element (e.g., a protein, enzyme, antibody, or receptor) [114]. Electrochemical biosensors are normally robust, sensitive, easily miniaturised, generate rapid output signals, have low detection limits, and can be made for field analysis and point-of-care clinical applications [115]. Based on their types of measurement, electrochemical biosensors are classified into three categories: (a) current (voltammetric and amperometric), (b) potential difference (potentiometry), and (c) impedance (electrochemical impedance spectroscopy).

Amperometric sensors are based on the measurement of an electrical current that results from either electrochemical oxidation or reduction of the electroactive species in a biochemical reaction, where the potential (voltage) across the sensor is kept constant during the measurement [116]. However, complex voltage wave forms can be used in other configurations to detect multiple active analytes. Common examples of this type of sensor include the amperometric measurement of oxygen and enzyme-based sensors, which are capable of transducing the rate of a biochemical reaction into a measurable signal [117,118]. Potentiometric biosensors are based on the determination of the potential difference between the sensor and a reference electrode. Potentiometry depends on the equilibrium state created by differences in ion concentration, which bind selectively at the electrode surface and explicitly provide the information about differences in ionic concentration [119]. For example, field-effect transistor-based potentiometric devices are widely in use to measure pH, and relative ionic concentrations in solution [120]. Combinations of optical and electrochemical techniques have been developed recently and for a wide range of applications where the effect of photovoltaic is utilised to determine the potential distribution along the interface of the sensor and this is known as a Light Addressable Potentiometric Sensor (LAPS) [121]. Impedimetric biosensors measure changes in the interfacial resistance and capacitance of the material at the chemically modified electrode surfaces which is due to the small amplitude sinusoidal AC excitation signal [122]. Therefore, impedimetric detection is primarily used for affinity type biosensors for example to monitor the immunological binding of antibody-antigen (Ab-Ag) on the sensing surface. Also, graphite-based impedance sensors for procalcitonin have been developed that can be used as a marker for sepsis.

**Figure 2:**
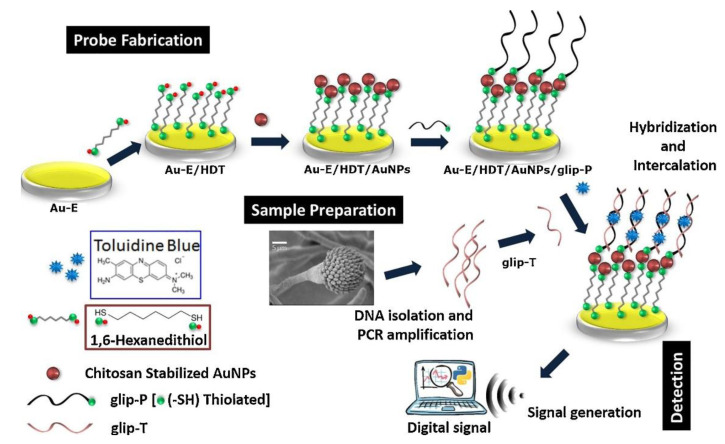
Fabrication and detection principle of glip biosensor. Initially, 1,6-hexanedithiol (HDT) is self-assembled onto a gold electrode (Au-E) to which chitosan-stabilised AuNPs (gold nanoparticles) are linked to the Au-E/HDT surface. Next, thiolated glip probes (glip-P) are allowed to react and are thereby immobilised onto the Au-E/HDT/AuNPs probe. Finally, the detection of the virulent glip target gene (glip-T) is achieved by hybridization followed by intercalation of toluidine blue that provides the analytical signal. Reprinted with permission from [124].

Despite the potential and multiple advantages of electrochemical methods, a limited number of publications have so far reported on their application for fungal diagnosis. Direct detection of *C. albicans* has been achieved using membrane based electrochemical impedance spectroscopy [123]. A sensor electrode primed with anti-*Candida* antibodies appeared to have a detection sensitivity of 10 CFU/mL. However, the method required further modification and optimization so has yet to be applied to the detection of *C. albicans* in clinical samples. Electrochemical biosensors have been designed to detect *A. fumigatus* using chitosan-stabilised gold nanoparticles [124]. The sensor probe was constructed using multiple fabrication steps, as depicted in (Figure 2), in which toluidine blue was employed to generate the electrochemical signal. Asghar and colleagues (2019) reported a novel immuno-based microfluidic device for the rapid detection of *C. albicans* in human whole blood [125,126]. However, disappointingly, the microchip technology suffered from low sensitivity and was only able to capture *C. albicans* in phosphate buffer solution with an efficiency of 61–78% for cell concentrations ranging from 10 to 10^5^ CFU/mL. However, the principle is portable, adaptable and has the potential to be refined to give higher levels of specificity and sensitivity. Graphene was hailed as a novel material for the construction of electrodes for electrochemical sensors giving a wider operating voltage range and relative freedom from poisoning in a hostile biological milieu. This may be important for the future construction of sensors that could be implanted into patients.

### 10.2. Optical Biosensors

**Figure 3:**
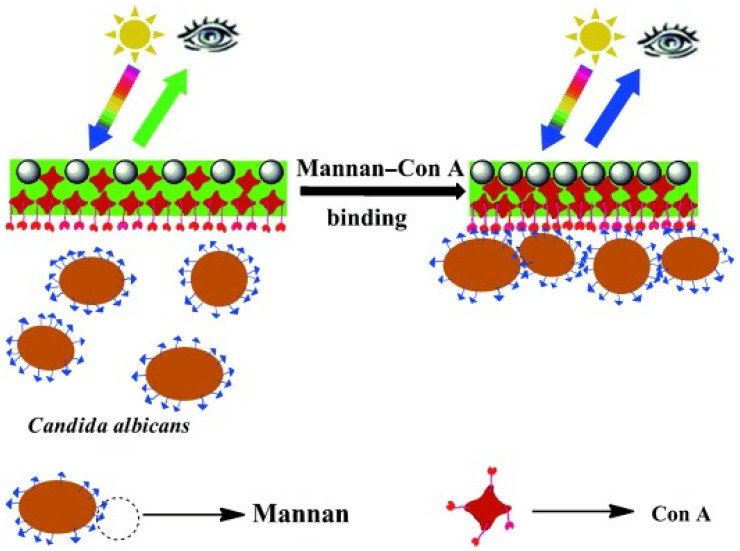
Schematic illustration of a sensor of mannans—a major surface carbohydrate of *C. albicans*. 2D photonic crystal (PC) arrays were synthesised using monodisperse polystyrene (PS) spheres. Thereafter, concanavalin A (Con A) mannose-binding lectin hydrogel was prepared by mild crosslinking of a Con A monomer solution with glutaraldehyde on the surface of the 2D PCs. The hydrogel Con A proteins each bind multiple mannose groups to surface-crosslink the protein hydrogel, resulting in shrinking and a decrease in the 2D array particle spacing that results in a blue-shift of the 2D array diffraction indicating the detection of mannans. Reprinted with permission from [130].

Optical-based biosensors are compact analytical devices containing a bio recognition element integrated with an optical transducer [125]. The working principal of such biosensors is based on the intensity of adsorbed or emitted light that is proportional to the change in the physical quantity of a measured analyte, where the refractive index (RI) of the medium is changed due to the interaction between molecules in the analyte and receptors present on the substrate`s surface. The change in RI is directly related to the amount of analyte in the sample, thus providing a quantitative measure of the target analyte which, providing it is sufficiently sensitive and reactive, could be equated with burden of infection or response to treatment. Optical biosensors also provide useful information in regard to the affinity of the receptor, and association and dissociation kinetic interactions [127]. These have advantages over other analytical techniques as they are capable of making real time measurements and can generate label free detection of many chemical and biological substances. They have been effectively exploited in multidisciplinary fields such as microelectronics, molecular biology, chemistry, and microelectromechanical systems [128,129]. To date, optical-based fungal biosensors have not been explored in detail; however, Cai and colleagues (2015) used a photonic crystal sensor based on cell-surface mannan binding to hydrogel Con-A [127]. The cross-links that were formed shrink the Con A hydrogel volume and decrease the 2D array particle spacing and the system was able to detect 32 CFU/mL (Figure 3).

Many of the optical biosensor platforms are based on the use of highly versatile and ultrasensitive transducers [131] which could be used for the detection of fungal biomarkers. One example of such a platform is the micro-optical biosensors called whispering gallery mode (WGM) biosensors. WGM biosensors have already been investigated for applications in biosensing that range from the detection of molecules to the detection of bacterial cells [132]. The glass WGM sensors could be modified with molecular receptors such as lectins, immune receptors and antibodies, to facilitate the specific detection of fungal biomarkers. Furthermore, they could be combined with some of the nanoengineered structures listed in Table 1. Already, the combination of WGM biosensors with plasmonic nanoparticles has improved the detection limits of the WGM platform. The resulting optoplasmonic sensors have been used for the ultrasensitive detection of single molecules [133] and the direct detection of glucosidase enzyme activity at the single-molecule level (S. Subramanian et al., submitted, 2020).

### 10.3. Piezoelectric Biosensors

Piezoelectric biosensors are a group of analytical devices based on affinity-based interaction recording. A piezoelectric platform or piezoelectric crystal acts as the sensor. The oscillations of the piezoelectric surface changes upon binding of the analyte on the piezoelectric crystal surface [134,135]. The analytical signal is measured as a change (decay) in oscillatory frequency, which is proportional to the mass bound to the crystal. Despite the fact that piezoelectric biosensors would be an excellent choice for label-free determination of an analyte and the fact that this area of research has great potential, to our knowledge there are no reports to date of such techniques applied to fungal detection.

### 10.4. Thermal biosensors

The working mechanism of a thermal biosensor is based on the measurement of the heat change in a medium that occurs as a consequence of a biochemical reaction. This change in heat is used generate an electronic signal [136]. Again, to our knowledge, there are no reports of thermal biosensor methodologies being investigated in the context of fungal diagnostics.

## 11. Emerging Diagnostic Methods

Currently, most fungal diagnostic methods require invasive sampling, and/or are time consuming, and/or are limited by their specificity or sensitivity. Hence, the development of new analytical methods that address these limitations is of importance in developing enhanced fungal infection management strategies. Advancement in the field of nanotechnology has allowed the combination of multiple analytical methodologies to establish new possibilities in this domain. For example, spectrophotometric approaches have been used to detect *Paracoccidioides brasiliensis* [138], and more recently a combination of artificial intelligence and metabolomics was reported for paracoccidioidomycosis [139]. Fluorescence in situ hybridization methods have been described for *Aspergillus* and *Candida* species [140,141]. Matrix-assisted laser desorptionionization time of flight (MALDI-TOF) analysis now provides highly accurate and rapid identification strategies for *Candida* and other yeast species which are rapidly being adapted for filamentous fungi [109,142]. This technique is also being evaluated for its utility as a diagnostic method on primary patient samples. This is, however, an expensive option that requires specialist technical knowledge and equipment and cannot be readily applied in field situations. Data bases containing information of mass spectrometry fingerprints are expanding but are not always reliable and universally available.

Recent research has also focused on the synthesis of novel nano materials for the construction of analytical platforms to improve sensitivity and specificity. Such materials include nanoparticles (NPs) made from gold or silver that can be utilised for different purposes, including biomolecule immobilization, signal amplification and target recognition [143]. Other nano-size materials that have been employed in various bioassays include quantum dots, molecular beacons, DNA dendrimers, carbon nanotubes, liposomes, nanowires and nucleic acids [144] (Table 2).

More recently, microfluidic-based methods have become an active research area for fungal detection [145]. There have been published reports based on different mechanisms of detection, for example. *C. albicans* DNA was detected in human blood utilising a real-time PCR-based microfluidic platform [146], with other reported methods including the use of AuNPs, peptide nucleic acid, Au-nanowire, nanoparticles and colloidal gold and silver [147,148,149,150]. Recently, a polymerase free assay was reported for *C. albicans* detection in clinical samples [151]. The proposed method was based on Single MOLecule Tethering (SMOLT), displacement of the beads tethered by DNA probes generated the signal, where the proposed method was able to detect 1 CFU/mL in blood sample. A colorimetric method for the detection of *Aspergillus niger* spores was developed based on interactions between fungal spores and gold nanoparticles modified with a specific binding peptide that was identified by phage display screening. The specific binding peptides were able to detect 50 spores within 10 min [152]. As it stands, this will have limited clinical utility as fungi rarely spore in vivo; however, it could be adapted to detect hyphal wall components.

## 12. Current and Future Fungal Biosensors and Biomarkers

There are two requirements for the fabrication and development of fungal biosensors for clinical use: (i) specific biomarkers have to be identified that would enable specific detection of the target organism, preferably from a range of clinical samples. (ii) The selected biomarkers must be able to be immobilised effectively onto the sensing surface.

Biomarkers must have characteristics that can be measured and used to indicate a normal or pathogenic condition. The detection of biomarkers can be either qualitative providing digital (yes/no) or quantitative above a threshold normal level defining the relative burden of infection. Biomarkers can be cellular or molecular in nature (DNA, RNA, protein, metabolites) and can be measured in tissue biopsies or in biological fluids (CSF, blood, urine, etc.). Physiological, morphological biomarkers can also be used or measured using clinical or medical imaging. A wide range of fungal biomarkers have been explored as candidates for the development and, as previously described, detection of some biomarkers, such as galactomannan, mannan, beta-glucan and cryptococcal antigen, which are in routine clinical use [10,154] (see above). These biomarkers which have already been recognised and developed into diagnostic tests could be investigated for their potential adaptation for detection via a biosensor mechanism, which could then be miniaturised and incorporated into a portable device. Other biomarkers as described below have potential but have not as yet been developed into diagnostic tools.

## 13. Pattern Recognition Receptors

The immune system is armed with a wide range of pattern recognition receptors (PRRs) that specifically bind to pattern-associated molecular patterns (PAMPs), which are mostly structural components of the outer cell walls and layers of pathogenic microorganisms. Because some of these PRRs are capable of binding specifically to fungal structures, they have significant potential in the development of fungal-specific biosensors. Within the PRRs, C-type lectins (CTLs) are a class of receptor that binds to carbohydrate structures, and CTLs exist that bind to β-1,3-glucan, chitin and fungal mannans [155,156].

For example, dendritic cell-associated lectin-2 (dectin-2) is a C-type lectin family that is known to bind fungal mannan [157]. This protein is most well characterised, encoded by six exons and has a single carbohydrate recognition domain in the extracellular, stalk, transmembrane regions, and a short cytoplasmic domain with no known signalling motif. Dectin-2 protein binds to high-mannose structures [158], and, in *C. albicans*, the outer cell wall *N*-linked outer-chain mannan fibrils are recognised [157]. Binding by dectin-2 leads to a series of cellular responses initiated by dectin-2, such as the release of inflammatory cytokines and production of reactive oxygen species. The possible utility of dectin-2 as a biosensor in nano particle-based biosensor is now being explored [159].

## 14. Siderophores

Siderophores (iron carrier) are low-molecular-weight Fe^3+^ chelating molecules produced under iron-depletion conditions that transport iron across the cell membrane. Iron acquisition by siderophores is an energy-dependent mechanism. Fungal siderophores identified so far include the hydroxamate type that can be classified into three structural families: fusarinines, coprogenes, and ferrichromes. Siderophore-detection-based diagnositics could potentially provide useful complementary information to confirm the presence of actively metabolising fungal cells and warrant further consideration and investigation [160].

## 15. Mycotoxins

Mycotoxins are toxic secondary metabolites produced naturally by many fungi under certain growth conditions that are capable of causing metabolic responses, disease or death in humans and animals. Although they have well recognised pharmacological actions, few mycotoxins or their derivatives have been categorised as antibiotics, growth promotants, and other kinds of drugs. Major mycotoxins include aflatoxins, gliotoxin, citrinin, ergot alkaloids, fumonisins, ochratoxin, and patulin. Although important, the details of mycotoxins and their detection have been reviewed extensively [161,162,163] and are beyond the scope of this review, which focusses on the detection of fungal cells. Their potential role as biomarkers of infection would also be limited by the fact that many are only produced under very specific growth conditions.

To date, none of these available biomarkers have generated sufficient sensitivity and specificity for the clinical diagnosis of fungal infections. However, the potential of using multi-biomarkers in a single diagnostic platform remains to be explored.

## 16. Biosensor Immobilization Techniques

**Figure 4:**
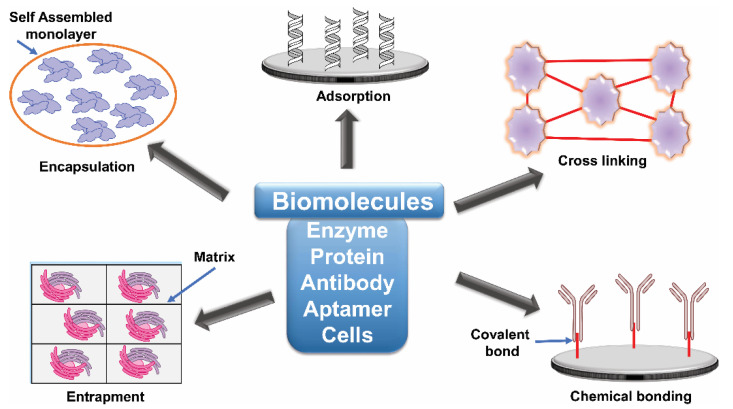
Immobilization methods for various biomolecules used in the construction of biosensors. Adsorption is a simple phenomenon where the biomolecules are attached through physical interactions generated between the carrier and enzyme through van der Waals forces, ionic interactions or hydrogen bonding. Glutaraldehyde is commonly used to cross-link enzyme molecules via the reaction of the free amino groups of lysine residues on neighbouring proteins. This is a desirable approach to achieve stronger immobilization than physical adsorption processes. Entrapment is the physical attachment of enzymes in a limited space. Matrix and membrane entrapment, including microencapsulation, are the two major methods used. Membranes include cellulose acetate, polycarbonate, collagen, and Teflon or other polymers. Encapsulation is used to avoid any negative influence on the structure of the enzyme, which uses a sol–gel, which is a chemically inert glass that can be shaped in any desired way. More recently, liposomes have been employed with bilayer-forming amphiphilic molecules, such as phospholipids.

An important step in the fabrication of biosensors is the immobilization and attachment of biomolecules to sensor surfaces. The sensors may be specific antibodies, aptamers, enzymes or whole cells, but, in each case, they need to be immobilised onto an appropriate surface. The process of immobilization must not affect the biological, physical and physiological activities of the biomolecule, and the precise method of immobilization must be considered as it can directly influence the performance of the biosensor. Immobilization methods must be efficient, and the costs must not compromise the efficient commercialization of the biosensors [164] (Figure 4).

### 16.1. Chemical Immobilization

Chemical immobilization occurs via either the addition of chemical cross-linking agents such as glutaraldehyde or direct covalent bond formation between the bioreceptor and a suitable surface [165]. Specific covalent bond formation can be achieved by interaction with cysteine amino acids present or introduced into surface loops of a protein biomarker and functionalised gold surfaces. A common example is based on glutaraldehyde as a cross-linking agent. This specifically forms a covalent bond with lysine residues on the surface of proteins. Covalent bonding is considered the most reliable and stable method for chemical immobilization, because it can improve the uniformity, density, and distribution of the bound sensor molecules, leading to increased sensitivity [166]. However, careful analysis must be undertaken during the chemical immobilization steps to ensure that there has been no loss of biological activity. Covalent linkages may use an activator such as 1-ethyl-3-(3-dimethylaminopropyl) carbodiimide (EDC) as a carboxyl activating agent for the coupling of primary amines to yield amide bonds or N-hydroxysuccinimide (NHS) converting carboxyl groups to amine-reactive NHS esters which can accelerate and control the immobilization process.

### 16.2. Physical Immobilization

Non-chemical immobilization methods can also be used where there is no chemical bond formation. The biomolecules are attached onto a suitable surface or matrix by adsorption, entrapment or encapsulation. Adsorption, mediated via Van der Waal’s, ionic and hydrophobic interactions, is perhaps the simplest option [167]. An advantage of this technique is that the immobilisation process is unlikely to alter the performance of the sensor molecule. However, the affinity of biological molecules to the surface may be low and may be influenced by changes in pH, temperature, and other factors. Therefore, the biosensors developed based on adsorption methods may suffer from a short shelf-life as well as reduced sensitivity and reproducibility.

Entrapment is based on the occlusion of the biomolecule, such as an enzyme, within a polymeric network that retains the biosensor molecule but is porous to the substrate and products [166]. Encapsulation is also a simple option to envelope, localize, or absorb the biomolecules into the sensor matrix surface. This can be achieved by either layer-by-layer deposition or by electrochemically polymerizing the polymer onto the surface within a mixed solution of enzyme and monomer of the polymeric matrix material. However, the practical use of these methods is often limited by mass transfer properties of the relevant materials through membranes or gels.

Immobilisation can also be achieved by the introduction of specific ‘tags’ on the protein of interest which can be introduced when making constructs for the over-expression of the recombinant enzyme. The most commonly used ‘tag’ is the His-tag, which can be introduced at the C or N terminus of the protein. This allows for a more gentle and potentially reversible immobilisation technique, where the histidine ‘tag’ has an affinity for a nickel support.

## 17. Characterization of Immobilised Biomolecules

The efficiency of immobilization of the biomolecule onto the desired surface can be confirmed and quantified by various characterization techniques. The orientation of such immobilised biomolecules requires that there is no disruption of the three-dimensional structure of the biomolecule whose properties are crucial for the stability and activity. Immobilisation must also not reduce the inherent conformational changes that occur during biomolecule function. Moreover, the molecular orientation of the biomolecule on the biosensor surface and the homogeneity of the surface coverage can play a critical role in the functionality of the biosensor.

## 18. Conclusions and Future Perspective

Fungal infections are an increasingly important health and economic burden. Accurate and early detection of fungal infections is essential to complement efforts in the development of therapeutic agents, since late diagnosis of fungal infections significantly compromises the likelihood that therapeutic interventions will be successful. Conventional diagnostic approaches have already made significant advances in our ability to identify and manage invasive fungal infections. The challenge is to accurately and rapidly detect the causative agent since this will often direct the appropriate antifungal management.

Current diagnostics will increasingly be augmented with the advance of nanotechnologies that can lead to the development of minimally invasive and miniaturised detection platforms. The next generation of biosensors are currently being developed using specific protein biomarkers immobilised onto gold nanoparticles. The detection of the biosensor interaction with specific targets on the surface of pathogenic fungi can be carried out using photonic techniques. These new methods offer considerable promise to tackle the problems associated with the rapid detection of fungal infection in human medicine.

Some of the emerging biosensor platforms that have already been used in other fields could be adapted to address the challenges in detecting fungal pathogens and fungal infections rapidly, with a high sensitivity and a high specificity. Biosensors could be used to develop highly sensitive, <100 pg/mL real-time testing capabilities for molecular biomarkers that are needed, i.e., for detecting *Candida* infections with a clinically relevant sensitivity. The composition of the fungal cell is an attractive target for developing the next-generation biosensor assays because its composition can be unique to the pathogen. Glucan biosensors could meet the demands for the high sensitivity when detecting cell wall fungal biomarkers associated with diseases such as the cell wall glucans β-(1,3)-glucan, β-(1,6)-glucan, mixed β-(1,3)-/α-(1,4)-glucan, and β-(1,3)-glucan. The typical serum glucan levels in subjects with candidemia [168] are on the order of 10^2^ to 10^4^ pg/mL. Biosensors could address the pressing need for fungal detection methods that are capable of detecting a panel of blood-borne fungal biomarkers so that targeted antifungal drugs can be administered rapidly to save lives. Combining different recognition elements on a multiplexed biosensor platform might enable us to record specific sensor signatures to achieve this goal [55,56,168]. Advances in biosensor research also offer the promise of generating implantable biosensors to continuously monitor fungal-specific and fungal-relevant analytes that would highlight critical points of intervention in intensive care settings. These opportunities and advances highlight the need for investment in diagnostic and biosensor research to tackle the problems associated with the rapid detection of fungal infection in human medicine.

## Figures and Tables

**Table 1:** Immunochromatographic tests for clinically relevant fungal species.

Type of Fungus	Assay Time	LOD or Sensitivity	References
*Aspergillus* spp.	15 min	37 ng/mL	[78,134]
*Cryptococcus neoformans*	10 min	>5 ng/mL	[137]
*Cryptococcus* spp.	5–15 min	100% (serum) 70.7–92% (urine)	[72]

LOD: limit of detection.

**Table 2:** Assays reported for fungal detection based on nanoengineered structures.

Fungal Species	Assay Time	Material	Sample	LOD or Sensitivity	Reference
*Paracoccidioides brasiliensis*	-	Au-NPs	Fungal DNA	>4 mg mL	[138]
*Candida albicans*	1 h	CNT	Fungal solution	50 CFU/mL	[153]
*Candida* spp.	30 min	Au-NPs	Waste water effluent	-	[149]
*Candida albicans*	2.5 h	PNA	Blood culture	100%	[147]
*A. fumigatus*, *C. glabrata*, *C. krusei*, *Cryptococcus neoformans*	A few hours	Au-nanowire	Fungal DNA	100 fM	[148]
*Candida* spp.	<3 h	Nanoparticles	Whole blood	1 CFU/mL	[150]

CNT, carbon nanotube; LOD, limit of detection; PNA, peptide nucleic acid; CFU, colony-forming units.
